# Supplementary material for: Brain connectivity fingerprinting and behavioural prediction rest on distinct functional systems of the human connectome
Source: Commun Biol. 2022 Mar 24;5:261. doi: 10.1038/s42003-022-03185-3 (PMC8948277; doi:10.1038/s42003-022-03185-3)
Supplement: Supplementary file 2 — Supplementary Information [file 42003_2022_3185_MOESM2_ESM.pdf]

SUPPLEMENT FIGURES AND TABLES

Supplementary Table 1: Correlations between all 30 behavioral measures

	FCCS	CCCS	TCCS	CF	gF	SA	GS	D	L/RD	LC	SO	ER
Fluid cognition composite	1	0.21	0.94	0.7	0.16	0.04	0.06	0.11	0.22	0.16	0.16	0.1
Crystalised cognition composite	0.21	1	0.51	0.17	0.21	-0.06	0.03	0.22	0.93	0.9	0.16	-0.01
Total cognition composite	0.94	0.51	1	0.66	0.2	0.01	0.06	0.15	0.49	0.44	0.18	0.07
Cognitive flexibility	0.7	0.17	0.66	1	0.16	0.06	0.08	0.08	0.2	0.09	0.17	0.1
Fluid Intelligence	0.16	0.21	0.2	0.16	1	0.81	0.02	0.13	0.19	0.2	0.8	0.8
Sustained Attention	0.04	-0.06	0.01	0.06	0.81	1	0.02	0.03	-0.06	-0.04	0.83	0.94
Grip strength	0.06	0.03	0.06	0.08	0.02	0.02	1	-0.13	0.05	-0.01	0.03	0.03
Dexterity	0.11	0.22	0.15	0.08	0.13	0.03	-0.13	1	0.2	0.2	0.07	0.08
Language/Reading Decoding	0.22	0.93	0.49	0.2	0.19	-0.06	0.05	0.2	1	0.67	0.15	-0.03
Language comprehension	0.16	0.9	0.44	0.09	0.2	-0.04	-0.01	0.2	0.67	1	0.14	0.01
Spatial Orientation	0.16	0.16	0.18	0.17	0.8	0.83	0.03	0.07	0.15	0.14	1	0.81
Emotion Recognition	0.1	-0.01	0.07	0.1	0.8	0.94	0.03	0.08	-0.03	0.01	0.81	1

Supplementary Table 2: Results for all 30 behavioral predictions for positive and negative models

Behaviour	Correlation Positive model	*p value*	MSE positive model	Edges Positive model	Correlation Negative Model	*p value*	MSE negative model	Edges Negative model	Subjects (n)	Adjusted *p* positive	Adjusted *p* negative
Fluid cognition composite	0.22	< .001	136.97	1298	0.25	< .001	133.74	1242	318	.002	< .001
Crystalised cognition composite	0.21	< .001	100.82	754	0.16	.005	105.53	648	320	.001	.028
Total cognition composite	0.25	< .001	208.57	1198	0.27	< .001	203.76	1162	318	< .001	< .001
MMSE	-0.06	.277	1.38	312	-0.01	.842	1.34	304	320	0.79	1
Cognitive flexibility	0.18	.001	104.89	1522	0.21	< .001	103.61	1746	319	.006	.002
Inhibition	0.12	.028	111.43	678	0.06	.318	118.18	538	320	.12	1
Fluid Intelligence	0.22	< .001	22.4	798	0.15	.008	23.72	528	319	< .001	.045
Processing Speed	0.14	.015	261.23	896	0.18	.001	252.13	1010	320	.071	.01
Working Memory	0.09	.102	136.68	730	0.16	.004	129.47	864	320	.307	.022
Sustained Attention (Sensitivity)	-0.01	.855	0.004	276	-0.02	.783	0.004	232	319	1	1
Sustained Attention (Specificity)	0.17	.003	0.001	636	0.12	.026	0.001	692	319	.015	.105
Personality: Agreeableness	0.1	.077	38.83	520	0.07	.236	39.26	556	319	.256	.786
Personality: Openness	0.01	.893	44.46	382	-0.04	.51	45.62	262	319	1	1
Personality: Conscientious-ness	0.03	.584	35.58	332	-0.04	.466	37.14	374	319	1	1
Personality: Neuroticism	0.05	.392	63.47	392	0.07	.206	61.98	496	319	.979	.728
Personality: Extraversion	0.05	.357	41.11	348	0	.978	42.68	278	319	.931	1
Grip strength	0.44	< .001	101.09	2534	0.42	< .001	104.07	2106	319	< .001	< .001
Dexterity	0.23	< .001	121.06	1230	0.21	< .001	124.96	1176	320	< .001	.002
Audition	0.06	.306	2.42	348	0.05	.357	2.46	364	317	.836	1
Smell	-0.12	.038	99.58	186	0.02	.714	92.96	296	319	.153	1
Taste	0.03	.637	236	532	0.12	.034	214.94	526	318	1	.126
Sleep Quality	0.03	.606	9.71	494	0.03	.628	9.76	556	320	1	1
Episodic Memory	0.09	.104	189.55	476	0.17	.003	181.06	436	320	.307	.019
Language/Reading Decoding	0.19	< .001	126.58	632	0.14	.01	131.24	418	320	.003	.047
Language comprehension	0.19	< .001	88.69	752	0.17	.002	89.95	618	320	.004	.014
Delay Discounting 200	0.11	.053	0.05	524	0.03	.621	0.06	388	319	.198	1
Delay Discounting 40K	0.02	.692	0.1	412	0.05	.399	0.1	360	319	1	1
Spatial Orientation	0.21	< .001	19.11	914	0.16	.003	19.69	770	319	.002	.021
Emotion Recognition	0.2	< .001	7.08	1112	0.13	.02	7.56	942	319	.002	.086
Emotion Recognition (hits)	0.1	.062	156502.47	468	0	.995	168863.67	216	319	.218	1

Supplementary Table 3: SVR prediction and overlap analysis

Psychometric Variable	Prediction (r value); *p-value*	Predictive edges (n)	Edges Overlapping (n) with fingerprints	Mean ± SD of Permutation	*p-values*
Fluid Intelligence	.29; <.001	147	7	5.38 ± 2.14	.151
Language Comprehension	.34; <.001	155	6	5.54 ± 2.19	.310
Strength	.55; <.001	184	4	4.82 ± 2.02	.555

Supplementary Table 4: Fingerprinting accuracies in validation atlases

Parcellation Scheme	Percentage hits (R1 -> R2)	Percentage hits (R2-> R1)
Shen (main text)	96.7	97.3
HCP MMP 1.0	99.1	99,1
Brainnetome	96.8	97.3
AAL	90.9	91.2

Supplementary Table 5: Overlap analysis for all thresholds

Behaviour	Threshold	Model	N_edges Fingerprint	N_edges Prediction	Overlap	Mean overlap Permutation	SD overlap Permutation	*P values*
Fluid Intelligence	.001	neg	36	10	0	0	0	1
Fluid Intelligence	.001	pos	36	20	0	0	0	1
Language Comprehension	.001	neg	36	12	0	0	0	1
Language Comprehension	.001	pos	36	14	0	0	0	1
Strength	.001	neg	36	165	0	0.01	0.09	1
Strength	.001	pos	36	221	0	0.06	0.23	1
Fluid Intelligence	.005	neg	179	48	0	0.59	0.71	1
Fluid Intelligence	.005	pos	179	93	0	0.21	0.46	1
Language Comprehension	.005	neg	179	51	0	0.56	0.69	1
Language Comprehension	.005	pos	179	61	0	0.25	0.5	1
Strength	.005	neg	179	413	2	0.43	0.64	.496
Strength	.005	pos	179	531	1	1.15	1.02	1
Fluid Intelligence	.01	neg	358	109	1	2.58	1.36	1
Fluid Intelligence	.01	pos	358	185	0	0.71	0.82	1
Language Comprehension	.01	neg	358	103	1	1.35	1.11	1
Language Comprehension	.01	pos	358	140	0	1.2	1.04	1
Strength	.01	neg	358	667	8	3.33	1.73	.132
Strength	.01	pos	358	785	1	6.65	2.3	1
Fluid Intelligence	.05	neg	1789	552	24	39.63	5.34	1
Fluid Intelligence	.05	pos	1789	817	39	25.92	4.37	.072
Language Comprehension	.05	neg	1789	568	41	42.31	5.27	1
Language Comprehension	.05	pos	1789	717	32	41.46	5.34	1
Strength	.05	neg	1789	1738	83	77.84	7.63	1
Strength	.05	pos	1789	2106	113	107.58	8.35	1

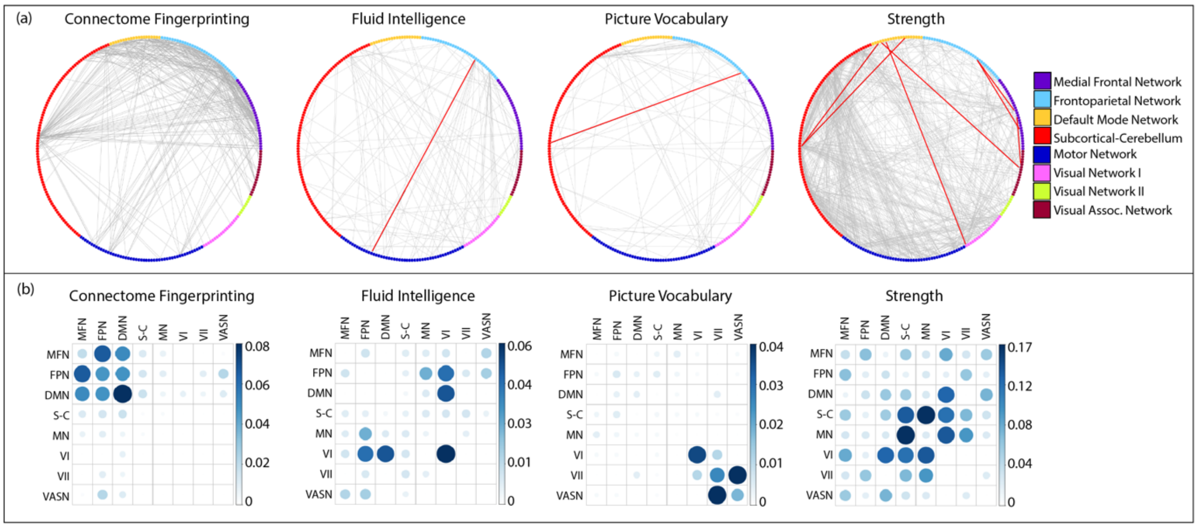

Supplementary Figure 1: Single-edge and between-network overlap for fingerprints and the negative prediction models, all thresholded at the 99th percentile.

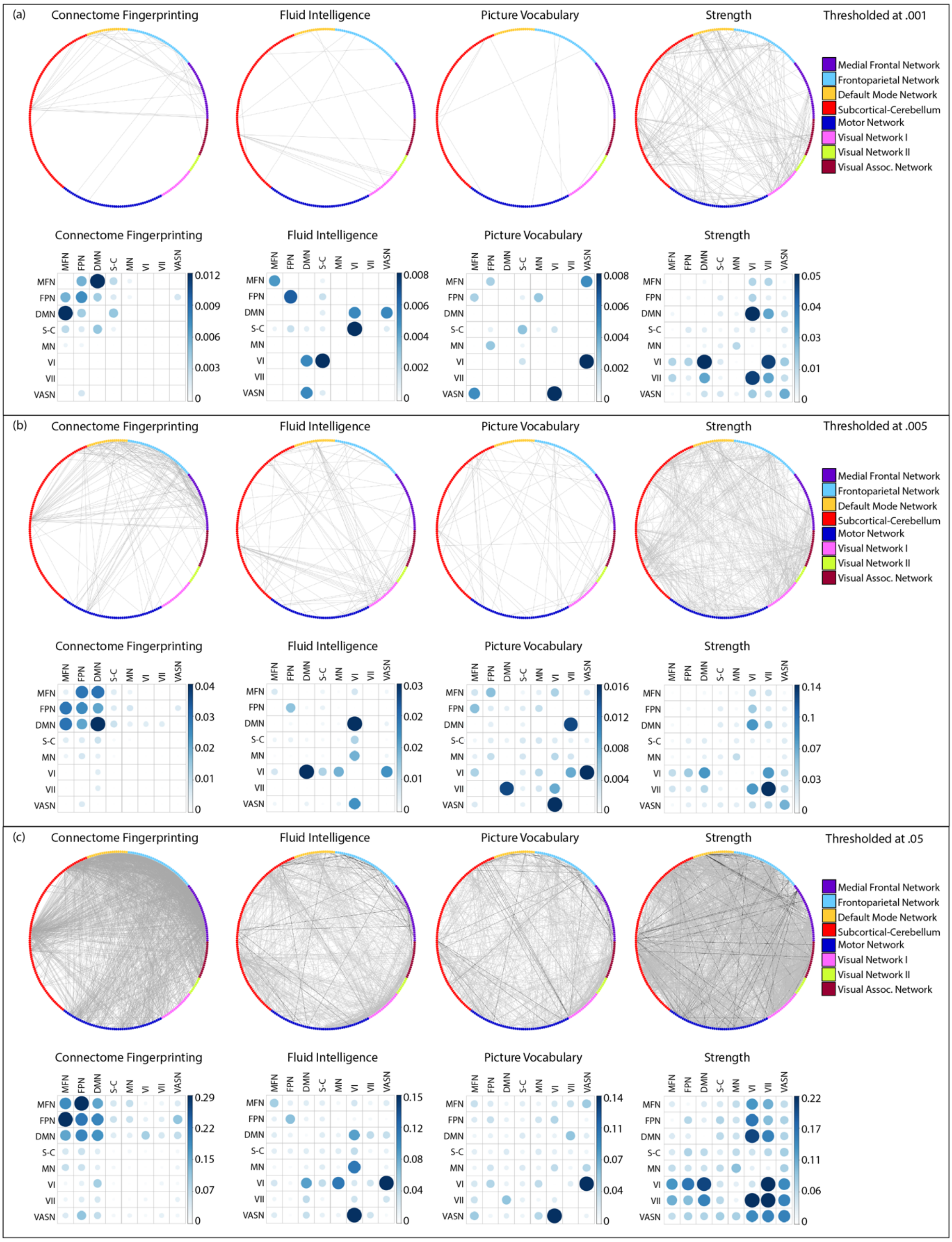

Supplementary Figure 2: Single-edge and between-network overlap for fingerprints and the positive prediction models, thresholded at the 99.9th, 99.5th and 95th percentile.

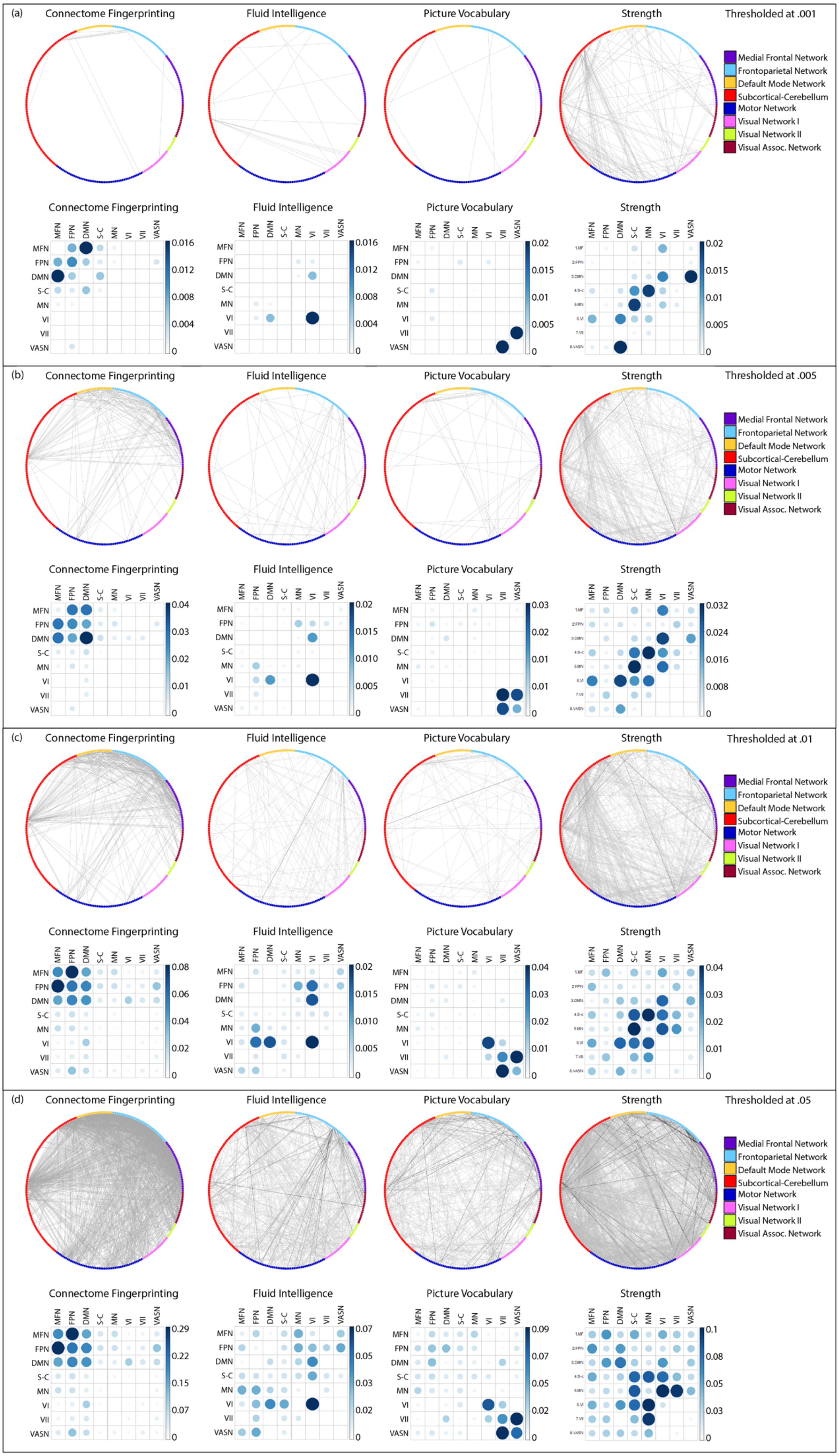

(a)
Connectome Fingerprinting
Fluid Intelligence
Picture Vocabulary
Strength
Thresholded at .001
Medial Frontal Network
Frontoparietal Network
Default Mode Network
Subcortical-Cerebellum
Motor Network
Visual Network I
Visual Network II
Visual Assoc. Network
MFN
FPN
DMN
S-C
MN
VI
VII
VASN
0.016
0.012
0.008
0.004
0
0.02
0.015
0.01
0.005
(b)
Thresholded at .005
0.04
0.03
0.02
0.01
0.010
0.032
0.024
0.016
0.008
(c)
Thresholded at .01
0.08
0.06
0.04
(d)
Thresholded at .05
0.29
0.22
0.15
0.07
0.05
0.09
0.1

Supplementary Figure 3: Single-edge and between-network overlap for fingerprints and the negative prediction models, thresholded at the 99.9th, 99.5th, 99th and 95th percentile.
