# Supplementary material for: Brain connectivity fingerprinting and behavioural prediction rest on distinct functional systems of the human connectome
Source: Commun Biol. 2022 Mar 24;5:261. doi: 10.1038/s42003-022-03185-3 (PMC8948277; doi:10.1038/s42003-022-03185-3)
Supplement: Supplementary file 3 — Description of Additional Supplementary Files [file 42003_2022_3185_MOESM3_ESM.pdf]

Description of Additional Supplementary Files

File name: Supplementary Data 1
Description: Source data underlying Figures 1a, 4b, and 5d.
