# Supplementary material for: Brain connectivity fingerprinting and behavioural prediction rest on distinct functional systems of the human connectome
Source: Commun Biol. 2022 Mar 24;5:261. doi: 10.1038/s42003-022-03185-3 (PMC8948277; doi:10.1038/s42003-022-03185-3)
Supplement: Supplementary file 5 — Reporting Summary [file 42003_2022_3185_MOESM5_ESM.pdf]

nature research

Corresponding author(s): Maron Mantwill

Last updated by author(s): Dec 7, 2021

Reporting Summary

Nature Research wishes to improve the reproducibility of the work that we publish. This form provides structure for consistency and transparency in reporting. For further information on Nature Research policies, see our Editorial Policies and the Editorial Policy Checklist.

Statistics

For all statistical analyses, confirm that the following items are present in the figure legend, table legend, main text, or Methods section.

n/a	Confirmed	
☐	☒	The exact sample size (n) for each experimental group/condition, given as a discrete number and unit of measurement
☐	☒	A statement on whether measurements were taken from distinct samples or whether the same sample was measured repeatedly
☐	☒	The statistical test(s) used AND whether they are one- or two-sided<br>*Only common tests should be described solely by name; describe more complex techniques in the Methods section.*
☒	☐	A description of all covariates tested
☐	☒	A description of any assumptions or corrections, such as tests of normality and adjustment for multiple comparisons
☐	☒	A full description of the statistical parameters including central tendency (e.g. means) or other basic estimates (e.g. regression coefficient) AND variation (e.g. standard deviation) or associated estimates of uncertainty (e.g. confidence intervals)
☐	☒	For null hypothesis testing, the test statistic (e.g. F, t, r) with confidence intervals, effect sizes, degrees of freedom and P value noted<br>*Give P values as exact values whenever suitable.*
☒	☐	For Bayesian analysis, information on the choice of priors and Markov chain Monte Carlo settings
☒	☐	For hierarchical and complex designs, identification of the appropriate level for tests and full reporting of outcomes
☐	☒	Estimates of effect sizes (e.g. Cohen's d, Pearson's r), indicating how they were calculated

Our web collection on statistics for biologists contains articles on many of the points above.

Software and code

Policy information about availability of computer code

Data collection	Data was collected by the Human Connectome Project and accessed using ConnectomeDB.
Data analysis	We used custom code available at https://doi.org/10.5281/zenodo.4557011 as well as adapted the publicly available code by E. Finn which can be accessed online (https://www.nitrc.org/frs/?group_id=51). All analysis was performed with MATLAB and data visualization was done in R.

For manuscripts utilizing custom algorithms or software that are central to the research but not yet described in published literature, software must be made available to editors and reviewers. We strongly encourage code deposition in a community repository (e.g. GitHub). See the Nature Research guidelines for submitting code & software for further information.

Data

Policy information about availability of data

All manuscripts must include a data availability statement. This statement should provide the following information, where applicable:

- Accession codes, unique identifiers, or web links for publicly available datasets
- A list of figures that have associated raw data
- A description of any restrictions on data availability

Data is from the publicly available HCP repository and can be accessed at http://www.humanconnectomeproject.org/data/; all HCP participants gave informed consent (Van Essen, 2013). The list of unrelated subjects used here can be accessed at https://wiki.humanconnectome.org/display/PublicData/S900+Unrelated +Subjects+CSV

Field-specific reporting

Please select the one below that is the best fit for your research. If you are not sure, read the appropriate sections before making your selection.

☒ Life sciences ☐ Behavioural & social sciences ☐ Ecological, evolutionary & environmental sciences

For a reference copy of the document with all sections, see nature.com/documents/nr-reporting-summary-flat.pdf

Life sciences study design

All studies must disclose on these points even when the disclosure is negative.

Sample size	We used all 339 subjects from the unrelated sample from the HCP.
Data exclusions	In the prediction analyses, we excluded subjects with missing data for each behaviour separately.
Replication	We were able to reproduce our main findings using different selection thresholds (.001, .005, .01, .05), three additional parcellation schemes as well as different prediction algorithms. We also provide the subjects used in our analysis as well as detailled preprocessing steps and the scripts used, which should allow other researchers to arrive at our findings.
Randomization	Since our design did not require experimental groups, no randomization was performed
Blinding	In our design blinding was not a relevant feature, e.g. due to the lack of experimental groups.

Reporting for specific materials, systems and methods

We require information from authors about some types of materials, experimental systems and methods used in many studies. Here, indicate whether each material, system or method listed is relevant to your study. If you are not sure if a list item applies to your research, read the appropriate section before selecting a response.

Materials & experimental systems

n/a	Involved in the study	
☒	☐	Antibodies
☒	☐	Eukaryotic cell lines
☒	☐	Palaeontology and archaeology
☒	☐	Animals and other organisms
☐	☒	Human research participants
☒	☐	Clinical data
☒	☐	Dual use research of concern

Methods

n/a	Involved in the study	
☒	☐	ChIP-seq
☒	☐	Flow cytometry
☐	☒	MRI-based neuroimaging

Human research participants

Policy information about studies involving human research participants

Population characteristics	Our sample included healthy unrelated adults aged 22-35 (183 females, 156 males).
Recruitment	Not applicable (see Van Essen, 2012)
Ethics oversight	As we exclusively used data from the HCP, we relied on their ethics consortium from institutions in the US and Europe, i.e. the 'WU-Minn HCP Consortium'

Note that full information on the approval of the study protocol must also be provided in the manuscript.

Magnetic resonance imaging

Experimental design

Design type	Only resting-state fMRI was used
Design specifications	Subjects were tested twice in a span of 48 hours, each run is 14.4 minutes (1200 volumes, TR = 0.72)
Behavioral performance measures	We used 30 psychometric measurements found in the HCP repository (see supplement) with a focus on fluid intelligence, language comprehension and grip strength.

Acquisition

Imaging type(s)	Functional and strucutural during pre-processing
Field strength	3 Tesla
Sequence & imaging parameters	2 mm (isotropic) EPI + 0.7 mm (isotropic) T1W
Area of acquisition	Whole brain scans

Diffusion MRI ☐ Used ☒ Not used

Preprocessing

Preprocessing software	We used the HCP preprocessed data and additional preprocessing with CONN toolbox / SPM 12
Normalization	Normalization was performed using a single nonlinear transformation, according to HCP protocols.
Normalization template	Data was normalized to MNI space
Noise and artifact removal	Using CONN toolbox we regressed out 12 motion parameters (provided with the HCP dataset under Movement_Regressors_dt.txt), mean time courses of white matter, CSF and the global grey matter signal (approximating global signal).
Volume censoring	No volume censoring was performed.

Statistical modeling & inference

Model type and settings	Multivariate functional connectivity analysis using pearson correlation without GLM.
Effect(s) tested	We tested whether patterns supporting behavioural prediction and single subject identification overlap

Specify type of analysis: ☐ Whole brain ☒ ROI-based ☐ Both

Anatomical location(s) We used Brainnetome, HCP, Shen and AAL atlas.

Statistic type for inference (See Eklund et al. 2016)	Does not apply
Correction	FDR-correction was applied.

Models & analysis

n/a	Involved in the study
☐	☒ Functional and/or effective connectivity
☐	☒ Graph analysis
☐	☒ Multivariate modeling or predictive analysis

Functional and/or effective connectivity	Pearson correlation
Graph analysis	Using a binarized graph of the functional connectivity, we calculated the node degree
Multivariate modeling and predictive analysis	We used connectome-based predictive modeling (Shen et al., 2017; 10.1038/nprot.2016.178)
